# On Certain Sensitive Troubles of Mesocephalic Origin

**Published:** 1878-06

**Authors:** 


### Translation.

### ON CERTAIN SENSITIVE TROUBLES OF MESO-CEPHALIC ORIGIN.

By Dr. Couty (*Gazette Hebdomadaire*, Nos. 1, 3, 4, 1878).

(*Translated from the French by Lafayette W. Case, M. D.*)

We have studied in a former article (*Gaz. Hebdom.*, 1877, Nos. 30, 34, 36, 38) a variety of hemi-anæsthesia, qualified as *mesocephalic ;* and we have studied this morbid form apart, the better to show the existence of a new type of hemi-anæsthesia, differing by its anatomical and symptomatological characters from the types already known—cerebral and medullary. It seems necessary to us to now show that other sensitive troubles, anæsthesic or hyperæsthesic, of variable and complex forms, may be produced by other lesions of the mesocephalon ; and we shall see, moreover, that this second memoir completely confirms the deductions drawn from the facts contained in the former one.

### *I.—General Anœsthesia.*

We have indicated (loc. cit., p. 570) cases sufficiently numerous of lesions of the central portions of the pons varolii, seated more or less forward, in the superior and inferior layers, not having brought on any sensitive trouble ; from this we have concluded that the inter-crossing of the sensitive fibers in the pons varolii had no definite track, since whichever one of these median points of inter-crossing was destroyed, the sensitive functions remained intact, in consequence of a substitution. But other cases exist where lesions occupying the same median portions of the pons varolii have, on the contrary, determined considerable sensitive troubles, of *general anœsthesia ;* we will report briefly a few of them.

Case I. (Josias, *Thesis* of 1851, case II.) Insane woman of Charenton ; apoplectiform attack ; afterwards, contractions of the muscles of the face ; difficulty of pronunciation, but paralysis of the four limbs and *complete insensibility of both sides.*

Some hours after, possibility of movements of the limbs, and very obtuse sensation ; later, asphyxia, death.

*Autopsy.*—Hemorrhagic spot in corpora striata ; cerebral peduncles as if crushed and replaced by a mixture of blood and cerebral matter. If the pons varolii be cut, it is found replaced by a detritus of blood and nervous pulp, the effusion being equally pronounced to the right and left. Cerebellum and bulb intact.

The lesions have been less extensive in the following cases :

Case II. (Nunneley, *Union Méd.*, 1860, t. vii., p. 381.) Sixteen years ; loss of senses ; complete insensibility ; pupils equal, contracted, insensible to light ; paralysis incomplete of movements and of sensibility of the two sides of the body ; head hot ; elsewhere temperature natural. Death after seven hours.

*Autopsy.*—At the center of the pons varolii, but encroaching especially to the left side, a clot half the size of a walnut.

Case III. (Kirschberg, *Thesis* of 1835, case X.)—Loss of senses, intelligence, general sensibility abolished ; paralysis of four limbs ; pupils contracted ; pulse hard and slow ; face congested. Death fifteen hours after.

*Autopsy.*—In the center of the protuberance, hæmorrhage of the size of a small nut had made an irruption towards the cerebellar peduncles and in the fourth ventricle.

The thesis of Kirschberg contains two other cases of general anæsthesia ; case III, seat, center of pons ; case XI, right half of the pons and portion of inferior layers of the left half destroyed ; and we might report others ; that of Magnan, incomplete anæsthesia due to a hemorrhagic collection below the *processus cerebelli ad testes*, (*Soc. Anatom*, 1861) ; that of Cornil (*Soc. Anat.* 1860, p. 201) ; two others indicated by Longet (*Anat. et Phy. du Syst. nerv.*, t. i, p. 439), and also case I. of *Hayem's Memoir* on Basilary Emboli (*Archiv. de Phys.*, 1868, p 270), with a clot of one centimeter on a level with the origin of this artery, etc., etc.

But, in the majority of these cases, cases which it would be easy to multiply, the patient was paralyzed and comatose, so that the general insensibility may be regarded not as the direct effect of the lesion of the sensitive conductors, but as a trouble dependent upon the loss to the coma of the cerebral functions. To this objection we respond that in numerous cases reported in our first article, cases to which, we believe may be joined most of the cases of ordinary hemiplegia, the patients had retained their sensibility on one or both sides, although the paralysis or even the coma were complete, which clearly proves that these states, except at the latest period, are not sufficient to produce anæsthesia. We would respond especially in citing other cases of lesions of the pons analogous by their seat, and having determined also general anæsthesia, in spite of the complete absence of troubles of the intelligence, of the functions of the perceptive organ. Such are the following cases:

Case IV. (Bourceret, *Soc. Anat.* 1847, p 448).—In February 1845, the right half of the body became "griped" says the patient, always cold, and his limbs feebler; besides the patient squints and sees double. In May, he entered the hospital for a right pleurisy; strabismus diplopia, taste perfect, but general sensibility notably diminished; movements of limbs feeble and difficult, then left facial paralysis.

Still later the gait becomes uncertain and finally impossible, speech difficult, and this moment the insensibility is complete and general. Death, the end of June.

*Autopsy.*—At the posterior inferior part of the pons, at a level with its point of junction with the cord, a hard kernel the size of a nut, situated to the left but invading a little, the right half of the pons.

In this case there had been at the beginning alternate hemiplegia; this trouble so frequently connected as we have shown (*Gaz. Hebdom.*, 1877, p 602) with mesocephalic hemi-anæsthesia, may also co-exist, then, with general anæsthesia.

This general insensibility, without intellectual trouble has been also noted in four of the cases of lesion of the pons reported by Longet; we will cite only the following:

Case V. (*2nd of Longet's Syst. Nerv.*, t. i., p. 440.)

Woman; gait staggering as in drunkenness; then total loss of motility and sensibility. Excited; she felt nothing; placed on one side, she remained so until moved; she lost her sight completely without opacity of the corneas; she lost taste, hearing, smell, and life, in relation to external things; seemed extinct a month before her death.

*Autopsy.*—The posterior half of the mesocephalon has become scirrhous, lardaceous (unfortunately no exact localization).

We believe we should, on account of its symptomatological analogy, add to the preceding cases the following very clearly observed, and where the diagnosis, obstruction of the bulbar arteries, unfortunately not verified by an autopsy, has been made by a nervous pathologist of the value of M. Joffroy.

Case VI. (*Gaz. Méd.*, 1872, p. 494, and *Bulletin de la Société de Biologie.*) Man; 5th of March, right amblyopia; 20th of March, difficulty of swallowing without paralysis of movements; 22nd March, paralysis of left limbs, contraction of jaws; 24th March, incomplete left hemiplegia, drawing of patient to right; *cutaneous sensibility completely abolished* over the entire surface of the body; sense of taste normal; vision and hearing very much dulled on right side; lips, tongue immovable; difficulty of opening mouth and of swallowing; salivation; superior portion of face only movable; intelligence enfeebled; micturition involuntary; cardiac lesion. From the 1st to the 26th of April, anæsthesia disappeared; right eye and ear always feeble; left hemiplegia complete; right paresis without increase of temperature of limbs; paralysis. August 1st, notable amelioration, but always cold.

To this case of labio-glosso-laryngeal paralysis of apopletic form, as M. Joffroy has called it, we might join a very analogous case published in the *British Med. Journ.* and analyzed in Hayem's review (t. ix., p. 152); and also another of M. Hérard (*Union Médicale*, 1868), which is distinguished from the preceding ones by the absence of sensitive troubles.

Now how shall we make these cases where we see lesions of the pons, either median, central or medio-lateral, bring on general anæsthesia, agree with those reported in our former memoir, where lesions of the same points have not brought on any sensitive trouble? Shall we be content by proving simply that lesions of the median portions of the mesocephalon sometimes entail loss of sensibility and sometimes leave it intact? No, for in spite of their contradictory appearance, these two groups of cases complete, in confirming each other. Given, cases of hemi-anæsthesia due to a lateral mesocephalic lesion of the opposite side, and cases of median lesion of the pons without anæsthesic trouble, we have concluded that the sensitive conductors from the face, and even some of those of the trunk, cross each other in the mesocephalon, but without following a fixed course, each point of the intercrossing being capable of being supplied by its unaffected neighbors. Now we understand perfectly that if the lesion of the median portions of the pons be too extensive, of itself, or by consecutive phenomena of compression, the sensitive fasciculi crossing each other are either all affected, or those left intact are insufficient; and then there follows anæsthesia, not hemiplegic, as in cases of lateral lesion, but bilateral, general; this anæsthesia will rarely be complete, more often imperfect, as in cases I, II and IV, or tardy and temporary, as in cases IV and VI.

General anæsthesia is then possible in certain cases of extensive lesion of the median portions of the pons and bulb; but it seems capable of existing also in certain cases of unilateral lesion of the prolonged cord. We will only cite the following: general anæsthesia of the body, but not of the face, with deafness produced by hemorrhagic effusion having destroyed the inferior surface of the cerebellum, and extending upon the lateral parts corresponding to the medulla oblongata and pons varolii (Ollivier, case LV, of the thesis of Prevost, 1868), diminution of the sensibility, at first bilateral, in both hands, then unilateral of the left side, produced by a cavity of the right half of the bulb, at a level with its middle part and of its posterior layers, back of the olive in the restiform body and the intermediary fasciculus (*Hallopean, Soc. de Biologie*, 1869, p. 171); general anæsthesia from hemorrhagic effusion in the right inferior cerebellar peduncle (*Nonat. Gaz. Hebdom*, 1861, p. 57). M. Vulpian has also obtained general anæsthesia from unilateral lesion of the bulb in some of his experiments upon the origin of the bulbar nerves, for example, in Experiment I. (*Mémoires de la Société Biologie*, 1861).

This trouble of the sensibility is also somewhat frequently noticed in the thesis of M. Gouguenheim (*Anevrysme des Artères du Cerveau* 1866) in the cases which have relation to the arteries of the mesocephalon ; but one may conceive that a tumor as diffuse as an aneurism even when seated on a lateral artery, the vertebral, cannot be of great utility as regards pathogenesis.

The mechanism of these general anæsthesias from unilateral mesocephalic lesion, is easy to understand in the cases where, as in those of Ollivier and Hallopean, the conductors have been destroyed near the median line over a considerable extent ; it is evident, indeed, that the sensitive fibers, after having crossed each other on the median line, remain mixed, confounded, on each side, over a space of greater or less extent. But there are other cases, that of Nonat, for example, where the general anæsthesia is caused from a lesion of the inferior cerebellar peduncle, a conducting fasciculus which we have shown by a case (*Gazette Hebdomadaire*, 1877, p. 410), may be completely destroyed without producing any sensitive trouble, from which it results that, in the cases analogous to that of Nonat, the general anæsthesia, not being the direct and constant effect of the lesion, should be explained either by a mediate compression of a neighboring organ, or by one of those disturbing actions from a distance, usually called irritative, of which the mechanism has quite recently been so well exposed by Brown-Séquard (*Archives de Physiologie*, 1877, p. 411, 415).

Without insisting further we may conclude that *general anæsthesia* is nearly always *produced by a median lesion of the pons and bulb* (protubero-bulbaire), and we pass to another form of mesocephalic anesthesia not less interesting.

### *II.—Alternate Hemi-anæsthesia.*

We have described in our former memoir a variety of mesocephalic hemi-anæsthesia of one entire side, face and limbs ; and produced by a lesion of the pons or of a peduncle of the opposite side, the lesion being upon their external fasciculi and sometimes in less complete cases of their posterior fasciculi. We have reported these cases separately the better to demonstrate the existence of a mesocephalic type of hemi-anæsthesia, comparable as to localization of cutaneous sensitive troubles, with cerebral hemi-anæsthesia, and from these cases we have concluded that the sensitive fibers originating in the face intercross each other between the bulb and the pons. But let us suppose that the lesion of the pons, instead of being seated tolerably high up in the external fasciculi, be more extended in breadth, lower down, and that it invade the bulbar sensitive glands or their afferent fibers : then the anæsthesia will occupy the members and trunk of the opposite side ; but in consequence of the lesion of the glands or bulbar sensitive nerves, it will occupy the face of the same side ; the hemi-anæsthesia will be alternate, and this special form realized by the experimental physiologists (Vulpian, *Soc. de Biologie*, 1861, memoir cited ; Lussana and Lemoigne, *Archives de Physiologie*, 1877, p. 382), has been observed in several pathological cases.

Case VII. (Carré, *Gazette Médicale*, 1834, p. 569). Sick seven years. Muscles of left side of face paralyzed ; ocular, buccal, nasal and lingual mucous membrane of left side insensible when pricked with pen; skin of face sensitive on entire right side and to the left only on a level with and behind the auditory conduit; vision remains; audition enfeebled; buzzing in the left ear; smell and taste diminished on left side but not noticably so. The left side of the body is normal; the movements of the right side are incompletely paralyzed, and the sensibility there is almost extinct; speech is difficult. Later these symptoms become worse. Sudden death.

*Autopsy.* The left side of the annular protuberance affected in its entire thickness and considerably augmented in volume, and transformed into a black, lardaceous tissue; the nerves of the vicinity are more or less altered; the trifacial, the sixth pair, are confounded in the tumor; the facial, the acoustic, the glosso-pharyngeal, appear compressed and flattened. The cord and brain are intact.

Alternate hemi-anæsthesia has not been less clearly shown in the following cases.

Case VIII. (Brown-Séquard, *Jour. de Phys.*, v. i., p. 537, *Annam.*)—Twenty-eight years. Acute pain of right side of face, then loss of senses; complete paralysis of left arm, incomplete of left leg; anæsthesia of left side of body; right facial paralysis; anæsthesia of right side of face and of the ear; inflammation of right ear at the end of several weeks; difficulty of deglutition and mastication; deviation of tongue to the right.

*Autopsy.*—Semi-cartilagnous fibrous tumor of right side of pons, extending to the place of origin of the fifth pair in the pons covering the origin of this nerve and the entire right side of the pons, descending below this origin as far as the inferior third of the medulla oblongata; the surface of the root of the middle cerebellar peduncle, the pons at its level, the right anterior cord in contact with the tumor, etc., are softened, etc., etc.

Case IX. (Brown-Séquard, *Journ. de Phys.*, t. vii, p 631). Considerable diminution of motion and sensibility of the left members and right face; right conjunctiva vascular; voluminous cyst of right side of pons.

There appears also to have been alternate hemi-anæsthesia in this other case borrowed from M. Gubler.

Case X. (Case XI, of the first memoir of M. Gubler, *Gaz. Hebdomadaire*, 1876, p 752). Thirty-four years. In February, 1876, paralysis of sensibility and motion of left face; a little later, complete paralysis of sensibility and motility in the limbs of right side; the left limbs also appear troubled but in less degree; a little contraction to the right; phonation and deglutition difficult. Death.

*Autopsy.*—Several fibro-plastic tumors, one of which, situated in the thickness of the pons to the left of the median furrow, has produced softening all around; two others exterior on the same side of the pons varolii.

These four cases suffice for proving the existence of an alternate hemi-anæsthesia of mesocephalic origin, quite comparable with the alternate hemiplegia so well studied by M. Gubler: Anæsthesia of one side of the body and of the face on the opposite, an anæsthesia not only of the skin but of mucous membranes also, of the eye, inside of cheek, nose, of taste, and above all of audition on the same side, but which may spare the skin on a level with, and especially back of the ear, that is to say beyond the territory of the trigeminal. It appears to us useless then, to report other analogous cases, such as those indicated by Brown-Séquard, (*Journ. de Physiol.*, t. vii, p 307, 637); another more complete of Jodin, (*Journ. de Physiol. de Magendie*, 1826) with left hemiplegia and hemi-anæsthesia, complete deafness, paralysis of taste and smell; and finally one of Guillaume, where the course and topography of the anæsthesia were still more complex. (*Progrès Méd.* 1873, p 293.)

It will be remarked that all these cases except perhaps case IX, unfortunately very incomplete, have their point of departure in a neoplastic lesion, a tumor; perhaps we must conclude from this, that hemorrhagic effusions capable of determining this assemblage of symptons would be in an organ so important as the bulb, too extensive to be compatible with life, so that this symptom is only possible in lesions of a chronic course, as tumors. And the proof of the probability of this interpretation is that we shall find sudden lesions, but less diffuse, in still another form of mesocephalic anæsthesia still more limited, a veritable diminutive of the preceding; and we shall be able to draw from the preceding cases, anatomico-physiological deductions which can only be made after the study of this last form of mesocephalic anæsthesia.

### *III.—Limited Anæsthesias.*

The preceding facts show that a lesion invading at the same time the pons and the sensitive nuclei of the bulb as in case VIII, or, in the pons, both of the external commissural fasciculi and the emergent fibers of the bulbar nuclei as in cases VII, IX, X, determines an alternate hemi-anæsthesia; but suppose the lesion less extensive, situated further back and above on a level with the floor, and then, the sensitive commissural fibers not being invaded, we have the following symptoms:

Case XI. (Heydenreich, *Soc. Anatom.*, 1875, p. 131).—B., 24 years, syphilis for three years; for three months, vomiting; right eye but little movable; nomonymous diplopia; right side of face paralyzed; tongue slightly deviated to the right, flexible, flabby on this side; speech embarrassed, hesitating; sight remains, bottom of eye intact; *less sensibility of the right side of*

*the face*, intact in limbs, feebleness of right arm and leg, violent head-aches.

*Autopsy.*—Tubercular meningitis; caseous tumor, size of large hazelnut had hollowed out a lodgement at the expense of the pons and the superior portion of the bulb, resting on the superior part of the deformed and non-recognizable fourth ventricle, encroaching slightly upon the cerebellum.

In this case, the lesion was limited to the level of the floor of the fourth ventricle. In the following, it appeared to have been carried rather upon the fibers of the trigeminal in their course through the pons:

Case XII. (Stiebel, *Med. Chi. Trans.*, 1863, t. xlvi). Eleven years; bronchitis. June 23rd, headache, nausea; 25th, cephalalgia of left side, left ptosis, right angle of mouth deviated, left pupil dilated without visual trouble, no paralysis of extremities; 29th, head turned to the right; *ancæsthesia of left side of face*, constant cephalalgia; 30th, convulsions, death.

*Autopsy.*—Left cerebral peduncle at a level with the pons thickened and softened; it contains the opening of an abscess filled with pus, mixed with pulp-like cerebral substance. The abscess, perfectly circumscribed behind the pons, a point at the level of which is found the remains of a hemorrhagic clot, 90 lines in length, 40 in its greatest breadth; it leaves the origin of the sixth pair at a distance of one line from its internal limit.

The lesions seem to have been a little more diffuse in the following case:

Case XIII. (Stanley, cited by Brown-Séquard, *Journ. de Phys.*, t. ii., p. 130.) Cephalalgia, left hemiplegia of body, *left hemiplegia of face with corresponding loss of sensibility*, mucous membrane of left side of nose and left conjunctiva injected, hearing lost on left side, erysipelas frequent in the paralyzed portions of face.

*Autopsy.*—Tumor the size of a walnut in the left side of the pons, extending into the middle cerebellar peduncle at a level with the origin of the fifth and seventh pairs which it compresses.

Without reporting other cases more or less complete of bulbar lesions, having determined anæsthesia of the corresponding side of the face, for example that of Baeltz (case XLIV. of the thesis of Hallopean), case VIII. of Gouguenheim, another case abridged by Brown-Séquard (*Journ. de Phys.*, t. vii., p. 307), a case of Cassy and Lorreyte (*Bull. de la Soc. Anat.*, 1874), we would remind the reader that experiments have for a long time shown the reality of this facial hemi-anesthesia by a unilateral lesion of the bulb of the corresponding side. If cauterization of the floor practiced by Cl. Bernard, but superficially, has not appeared to induce any serious trouble (*Syst. Nerv.*, t. i, p. 296), M. Vulpian (Leçons sur le Syst. Nerv., p. 511), and long before Magendie, have proved that this facial hemi-anæsthesia takes place after a transversal hemi-section of the bulb, and they have explained it rightly by a lesion of the descending root of the trigeminal ; and Brown-Séquard, going further, has been able to determine facial anæsthesia in removing only the restiform body of the corresponding side (*Bull. de la Soc. de Biol.*, 1855, p. 337).

But the dissociation of symptoms may be still more complete ; and the anæsthesia from mesocephalic lesion still more limited.

It is so in Hallopean's case XLVIII., borrowed from Gubler ; in another case published by Bordier (*Gaz. des Hôp.*, 1866, p. 561), the patients presented with symptoms of labio-glosso-laryngeal paralysis, an anæsthesia very clearly limited to the velum palati, that is to say, to the glosso-pharyngeal.

In other cases, on the contrary, the anæsthesia has been limited to one sense—to the hearing.

Case XIV. (Voisin, *Soc. Anat.*, 1863, p. 486.)—Fifty-two years ; epileptiform attack, then loss of knowledge. The next day some movements were possible, but the speech was confused ; the tongue deviated to the right, the right side of the face paralyzed and the *hearing dull on this side.* The following days the intelligence returned completely. The limbs only simply paresic ; but always a right facial hemiplegia, with *deafness of this side.* Later, pleurisy, death.

*Autopsy.*—Tumor at the level of the convergence of the basilar and vertebral arteries, constituted by a clot the size of a goose quill. If section be made of the pons on a line with the point of emergence of the facial, softening exactly confined to the right half, limited by the intact peduncular fibers, prolonged by a slender point toward the anterior face, more widely toward the middle cerebellar peduncle and the fourth ventricle, without reaching its wall which appears intact. Total length, 25 millimeters; breadth, 15 millimeters.

From this case we may go to the following, reported by Brown-Séquard, unfortunately very incomplete:

Case XV. (Gairdner, *Journ. of Phys.*, vol. vii., p. 637.)—Difficulty of speech; left pupil dilated; imperfect vision (perhaps due to the pupillary dilatation) *and deafness.*

*Autopsy.*—Tumor the size of a pullet's egg in connection with the right cerebellar peduncle, the right half of the pons, of the bulb and of the cerebellum (that is to say evidently situated upon the posterior superior face of the pons and bulb).

These facts prove the importance of troubles of audition in mesocephalic lesions, an importance which however has been often noticed. It is thus that M. Lancereaux indicates the existence of auditory symptoms in a state of unique isolated sensitive derangement, as one of the frequent signs of obstruction of the arteries of this region (Lancereaux, thesis, 1862, p. 60), and to the preceding, may be also added numerous other cases of general paralysis, of ataxia, of sclerosis in patches, mentioned by Magnon (*Gaz. des Hôp.*, 1870), by Lionville (*Bull. de la Soc. de Biol.*, 1870), by Hayem (*Bull. de la Soc. de Biol. et Gaz. Méd.* 1876), by Pierret (*Revue Mensuelle*, 1877, No. 2), etc., etc.; cases in which one side of the face, one sense, the taste, the ear have become alone anæsthesic, often on one side only, as in the second case of Pierret, evidently as the result of a lesion also limited in the points of origin or in the mesocephalic track of the corresponding nerves.

If, then, sensitive troubles are lacking, as is known in the cases of systematic bulbar lesion, labio-glasso-laryngeal paralysis, descending sclerosis, etc., etc., it is seen that these troubles are, on the contrary, frequent in cases of more diffuse lesions, ramollissement, tumor or dissiminated sclerosis; they may affect forms the most various, and to sum up, anæsthesia produced by a lesion of the pons or bulb may be generalized, hemiplegic, alternate, or more limited, to one trigeminal, one glasso-pharyngeal, or one auditory nerve. But all these cases of alternate hemi-anæsthesia, or of anæsthesia limited to one side of the face, to one sense, anæsthesias due to a unilateral lesion of the pons or bulb, admit of other more important deductions.

We have seen in cases VII, VIII, IX, X, XI, XII, XIII, the facial sensibility disappear on the side where either the origin of the trigeminal or its emergent fibers have been injured; exactly as in the experiments of Magendie, Vulpian, Brown-Séquard, Lussana and Lemoigne, on hemi-section of the bulb; ablation of the restiform body, etc. Now from these cases, where a lesion situated either in the point of origin, as in case XI, or along the course of its emergent fibers, as in cases VIII, XII, XIII, has determined not a diminution of sensibility of the whole face, but a complete and unilateral anæsthesia *in the side corresponding to the lesion*, we conclude that the fibers originating from the point of origin of the trigeminal do not cross each other in their intra-bulbar portion; their course is direct from the point of origin to the corresponding side.

Experiments (Cl. Bernard, Syst. Nerv., t. ii; Vulpian, Leçons sur le Système Nerveux) have proved the bulbar origin of the auditory fibers without indicating their course; from these cases where we see, as in cases VII, VIII, XI, XIII, the hearing affected on the same side as the face, or alone, but on one side only, corresponding to a unilateral lesion of the pons and bulb, as in Voisin's case, should we not conclude that the fibres springing from the point of origin of the auditory nerve have also a direct course and do not intercross?

Without denying the existence of very intimate relations between two sensitive points of the bulb, of the same nature, we believe we may affirm that, given the preceding facts, these relations, at least as regards the trigeminal and auditory, are not established, as has been admitted, by an intercrossing of the emergent fibers: these emergent fibers go directly from the point of origin to the corresponding organ, and their lesion determines a unilateral anæsthesia of the same side.

Now, are these sensitive nuclei put in relation with each other by direct commissural fasciculi, analagous with those of which M. Vulpian has established the existence for the facial (*Bul. de la Soc. de Biol.*, 1861, in the memoir cited)? We may conceive this to be so, our experiments cannot enlighten us on this point; but it is sufficient for us to have established by facts this direct course of the sensitive fibers between the bulb and the face, a course which in our former memoir (*Gaz. Hebdom.*, 1877, p. 600) we had supposed to be demonstrated, intending to return to this point. And after having thus terminated the history, or rather the indication of certain forms of mesocephalic anæsthesia, we pass to another order of sensitive troubles not less curious ; *mesocephalic hyperæsthesias.*

The hyperæsthesic sensitive troubles to which Brown-Séquard long since called special attention, if we refer to the numerous cases we have gone over, appear to us more frequent in the cases of mesocephalic lesions than in analogous lesions of the brain ; and as with mesocephalic anæsthesias, they are also distinguished from hyperæsthesias of cerebral origin in that they may affect the most various forms.

### *IV.—General Hyperæsthesia.*

Hyperæsthesia may be general as in the following cases which we hastily sum up.

Case XVI. (Gobert, cited by Brown-Séquard, *Journ. de Physiol.*, t. i., p. 526.) A fall at seven years of age; since then unsteady gait, cephalalgia, smell perfect, sight diminished, especially in right eye, hearing rather more sensitive; spontaneous pains in limbs, at first on right side ; *sensibility exaggerated over whole body* ; paralysis of right limbs, then of left ; extremities cold, deglutition difficult ; death.

*Autopsy.*—On the median line, level with the vermiform process and fourth ventricle, a cystic tumor of the size of a hen's egg, compressing the left lobe of the cerebellum, having burrowed a deep lodgement in the peduncle of the same side, in the interior of the ventricle as far as the tubercular quadrigemina slightly atrophied ; pons deviated, etc.

The lesion and the symptoms were quite analogous in the following cases :

Case XVII. (Mignot, *Gaz. Hebdom.*, 1875, p. 827.) Incomplete amaurosis, deviation of the eyes, fixed occipital pain, *sharp*

42

*pains in the limbs, exaggerated venereal desire, more exalted cutaneous sensibility.*

*Autopsy.*—Hydatid cyst of cerebellum the size of an egg, incarcerated in the left lobe.

Case XVIII. (Laborde, *Soc. Anat.*, 1863, p. 343.) Fourteen years, staggering, tendency to fall backward, curvature of body to the right, left convergent strabismus, *lancinating erratic pains* in lower limbs, and *cutaneous hyperæsthesia*, vomiting, slight amblyopia.

*Autopsy.*—Tubercle the size of a walnut on the inferior surface of right lobe of cerebellum in the amygdala, compressing the origins of the pneumogastric and glosso-pharyngeals; a second tumor in the right half of the pons in its central portion.

The lesion appeared to have been still more limited, and in appearance still more limited to the cerebellum in the following case, unfortunately very incomplete, like the preceding, in an anatomical point of view.

Case XIX. (Andral, *Clinique*, t. iii., p. 716.) Pulmonary phthisis; rigidity of the head drawn back; limbs and senses unaffected, only *cutaneous hyperæsthesia*, on pressure and on motion; left side of face paralysed; conjunctiva red on this side.

*Autopsy.*—Tubercle the size of a hazelnut on the middle portion of the external face of the left lobe of the cerebellum.

These diverse observations and especially the last, would tend to lead us to regard general hyperæsthesia as a trouble of cerebellar origin; but in the first cases the cerebellar lesion was seated at a level with the fourth ventricle, more or less compressed, as shown by the autopsy; and given symptoms of direct facial hemiplegia, inflammation of the conjunctiva of this side, it is probable that even in Andral's case, the bulb, the origins of the facial, the trigeminal, were also interested. We are then lead to explain these cases of general hyperæsthesia, following a cerebellar lesion; not by this lesion itself, but by consecutive troubles of the pons; and we shall see that general hyperæsthesia may indeed be due to a lesion occupying solely the floor of the fourth ventricle towards the union of the pons and the bulb, the cerebellum remaining intact.

Case XX. (Sandberg analyzed in *Hayem's Review*, t. ix).—

Notable feebleness of certain muscles; oblique gait; passing paralysis of right arm ; abdominal pains, *cutaneous hyperæsthesia.*

*Autopsy.*—Pia mater thickened in certain points, especally towards the base ; hemorrhagic clot having destroyed the floor of the fourth ventricle with extravasation into its cavity.

Case XXI. (Extracted from thesis of Hallopean, case XXXV, p. 108).—Sixty-three years. July 5th, vomiting; impossibility of swallowing and of standing, then incomplete paralysis; intelligence intact. July 6th, he entered service of M. Proust, trembling of hands very marked ; patient staggers, totters, especially left side; *hyperæsthesia* of lower limbs. The next day, sudden death.

*Autopsy.*—Arteries especially in encephalon, atheromatous; left vertebral artery rigid, obstructed one centemeter from its junction with the basilary, by a discolored, yellowish clot, one centimeter in length ; but leaving free the postero-inferior cerebellar which originates rather low down.

These pathological cases would perhaps be insufficient if we could not join to them experimental facts. Brown-Séquard has shown that section of the cerebellar peduncles, ablation of the restiform bodies, all lesions occupying only a level with the fourth ventricle, determine general hyperæsthesia (Brown-Séquard, *Bull. de la Soc. de Biol.*, 1855, p. 337); M. Vulpian has also obtained general hyperæsthesia in some of his experiments on the floor of the fourth ventricle (*Bull. de la Soc. de Biol.* 1861); and to sum up, in all cases of general hyperæsthesia cited above, there appears to have been always a lesion either direct or more often mediate and consecutive, of the floor of the fourth ventricle. We are then lead to conclude that this hyperæsthetic trouble with this generalized form is one of the symptoms of *lesions of this region of the floor of the bulb and pons varolii.*

It will also be remarked that, if in several of these cases, as in cases XVI, XVIII, XX, there has been staggering or curvature, a tendency to fall backward, etc., the real paralytic symptoms have been either lacking, as in cases XVII, XVIII, XIX, or very late, as in case XVI.

V.—*Hemi-hyperæsthesia.*

We will now analyze other cases, where the lesion, instead of being seated back of a level with the fourth ventricle, is situated higher up, in the antero-inferior layers of the pons; and with this difference in the location of the anatomical lesions we shall establish differences not less great in the sensitive or motor derangements. There will be hemiplegia, and not derangement of co-ordination, or general paralysis; there will be *hemi-hyperæsthesia*, and not general hyperesthesia. The concomitant hemiplegia has been most often alternate.

Case XXII. (Senac and Millard, *Gaz. Hebdom.*, 1856, p. 816).—March 18th, loss of knowledge, then hemiplegia of left limbs; right side of face and intelligence intact. The 20th, slight meandering of movements in left limbs, a little contracted; patient complains of *sharp pains* in paralyzed limbs; left forearm cannot be extended without sharp pain. The following days, amelioration, then new aggravation of symptoms, and death the 1st of April.

*Autopsy.*—Upon the inferior face of the annular protuberance to the left of the median line, a hemorrhagic clot the size of an almond had destroyed the pyramidal fasciculus to the extent of five millimeters, being prolonged toward the middle cerebellar peduncle.

We will only recapitulate the following cases:

Case XXIII. (Hillairet, *Soc. de Biol.*, 1860, p. 116).—Right facial paralysis; left limbs, sensibility rather exaggerated in left leg; pleurisy. Death.

*Autopsy.*—Central hemorrhagic clot, 2½ centimeters, had destroyed middle layer, and invaded inferior layer of pons.

Case XXIV. (Martineau, *Soc. Anatom.*, 1860, p. 311).—Former palpitations. July 7th, cephalalgia, creeping sensations on left half of body; incomplete hemiplegia of left limbs and right face; 8th, sensibility preserved and even exaggerated the slightest movement of *left* lower extremity causing the patient to cry out; later, pleurisy, then pericarditis. Death the 18th.

*Autopsy.*—Back of the tubercula quadrigemina, in the pons, sanguineous clot in the middle and lower layers, extending toward the right middle cerebellar peduncle, about two centimeters in diameter ; bulb and cerebellum intact.

Not to report other cases, unfortunately not followed by autopsies, and where the hemi-hyperæsthesia has also coincided with alternate hemiplegia, and with other symptoms surely of the pons ; cases due to M. Gubler (case V of his first memoir), to Meynert (analyzed in *Hayem's Review*, t. iii., p. 62) ; we will cite other examples of hemi-hyperæsthesia, with direct hemiplegia.

Case XXV. (Proust, cited by Déchery, Thesis, 1870).—Slight paralysis of left face, then complete of limbs of same side ; sensibility, instead of being diminished, increased in arm and leg. Insensibility of velum palati ; paralysis of pharynx and tongue.

*Autopsy.*—Clot in superior extremity of left vertebral, distending the artery and prolonged into the postero-inferior cerebellar.

There was also direct hemiplegia in the following case of Abercrombie, a case where the hemi-hyperæsthesia, although only complained of subjectively, appeared none the less real.

Case XXVI. (Longet, *Syst. Nerv.*, t. i., p. 446.) Thirty-seven years. March 23rd, diminution of respiratory amplitude on right side of thorax ; 25th, giddiness, numbness of entire right side, heaviness and creeping sensations of right limbs ; 30th, right ptosis, uneasiness, stiffness in the arm and leg of same side. Death.

*Autopsy.*—Cavity the size of a large hazelnut, filled with a clot, burrowed partly in the pons, partly between it and the cerebellum, most upon the left side.

Finally, the form of the hemiplegia did not appear to have been noted in the following observation, and also in another of Huguier (*Soc. Anatom.*, 1829, p. 53).

Case XXVII. (Josias, Thesis, 1867, case I.) April 6th, apoplectiform attack, then left hemiplegia ; tongue deviates to right : *sensibility, the state of which was investigated several times, appeared more acute on the side paralyzed.* Death the 23rd.

*Autopsy.*—Clot the size of a hazelnut, occupying right side of pons, near the anterior fasciculi, but somewhat distant from the bulb and the posterior fasciculi.

To sum up, hemi-hyperæsthesia is complained of by only one of the patients and after diverse excitations; or there is produced spontaneously, as in cases XXII, XXIV, XXVI, creepings, lancinating pains in the paralyzed parts: this hemi-hyperæsthesia has occurred tardily, as in cases XXII, XXVI; it has then persisted until death, but apparently diminishing in certain cases; or it has disappeared completely, as in the case of M. Gubler; or it has given place to hemi-anæsthesia, as in the case of Meynert. This hemi-hyperæsthesia occupies all one side of the body, the side opposed to the mesocephalic lesion; which differentiates it, as shown by Brown-Séquard, from hyperæsthesia by medullary hemi-section. The cases are too incomplete to say anything regarding the condition of the face or of the special senses.

In an anatomical point of view, the hemi-hyperæsthesia has been produced by a hemorrhagic lesion, more rarely embolic; this lesion always occupying the pons, unilateral, or at least the most considerable upon one side.

This unilateral lesion occupies the middle and anterior layers of the pons, as Cases XXII, XXIII, XXIV, XXVII prove; there is then a very clearly defined anatomical difference between this hemi-hyperæsthesia and general hyperæsthesia, which is always produced, we have seen, by a lesion of postero-inferior portions of the mesocephalon, by a lesion of the fourth ventricle, either primitive or consecutive to a cerebellar lesion.

Comparison of the two cases, XXI and XXV, both of M. Proust, indicates very well this difference of location, since we see an obstruction of the left vertebral determine general hyperæsthesia when it is situated one centimeter from the basilary, (Case XXI); and hemi-hyperæsthesia (Case XXV) when it occupies the origin of the basilary artery itself.

Hemi-hyperæsthesia also has anatomical characters very different from those of hemi-anæsthesia. Hemi-anæsthesia has often been caused by tumors, and we have not found a case of hemi-hyperæsthesia of neoplastic origin. Hemi-anæsthesia, in the cases where it has been complete, has been produced by a lesion extending by preference towards the posterior portions, and especially invading always more or less the lateral portions, the external fasciculi of the pons; now, in hemi-hyperæsthesia the lesion occupies on the contrary the anterior and middle layers, and above all this lesion always somewhat extensive, is central, median, internal and not lateral, as is expressly indicated in Cases XXII, XXIV, XXVI ; the effusion extends toward the middle cerebellar peduncle and not toward the superior ; it is situated to one side, within those external fasciculi, a lesion of which determines as we have shown in our first memoir, complete hemi-anæsthesia.

Brown-Séquard (*Journ. de Physiol.* t. i. p. 530, 760) has attributed this hemi-hyperæsthesia to an irritation of the gray matter of the pons. With Brown-Séquard, we admit in these cases an irritation ; but is this irritation one of the gray matter ? We have cited, in our first memoir, a large number of cases of median lesions of the pons, even extensive ones, not having brought on any sensitive derangement ; we have reported above, other cases of lesions still more extensive, situated especially in the posterior layer of gray substance, and having brought on general anæsthesia, because they destroyed all the sensitive decussating region not leaving a place for substitution ; now, we could not explain why lesions of the same nature, hemorrhagic, of the same gray matter, have determined in certain cases only, irritation with hyperæsthesia and, in others more numerous, negative or anæsthesic symptoms.

This first point being even admitted, from the fact that a unilateral irritation of a portion of the gray matter of the pons has determined a hyperæsthesia limited to the opposite side of the body, we should conclude that this gray substance has clearly localized functions ; and would not this conclusion be in disaccord with the numerous cases in which we have shown the possibility of a substitution between the diverse regions of the gray matter of the pons, in that which regards the sensitive functions.

In presence of these facts, especially if we remember that the lesion in hemi-hyperæsthesia, a lesion of the pons, anterior, interior and median, has extended towards the lateral parts into the middle cerebellar peduncle towards these external fasciculi, the lesion of which, we have shown, produces complete hemi-anæsthesia, we shall be forced to attribute this opposite hemi-hyperæsthesia *to an irritation*, from a clot situated in contact with them, *of these same external white fasciculi*, sensitive conductors of the peduncle and pons.

When the effusion destroys these postero-external fasciculi, there is opposite hemi-anæsthesia ; when the effusion, nearer the median line, does not affect these fasciculi except by inflammatory lesions, most often consecutive lesions of vicinity, there will be hemi-hyperæsthesia; and thus are explained the symptomatological particularities mentioned above ; the hemi-hyperæsthesia has always been produced by a hemorrhagic lesion, never by a tumor, the course of which has been too slow ; hemi-hyperæsthesia, most often, only occurs some days after the apoplexy, when the inflammatory irritation has had time to be produced ; the hemi-hyperæsthesia may diminish or cease with the irritation; finally, it is accompanied or preceded by formication, sharp lancinating pains in the limbs, and even, as in the case of M. Gubler, with veritable trifacial neuralgia, with neuralgia pains in the opposite limbs, with inflammatory swelling of the foot. There would veritably be in these hemi-hyperæsthesias, as in peripheric neuralgias, an inflammation of a white sensitive fasciculus ; only this fasciculus is commissural, myelencephalic ; the neuritis is central ; and thus this hemi-hyperæsthesia would enter into the class of neuralgias of central origin, a class much more numerous than is supposed, as M. Vulpian has quite recently shown. (Leç. sur les Mal. du Syst. Nerv., 1876, p. 4.)

We will now report two cases where the hyperæsthesia from an irritative lesion of the mesocephalon has been still more limited, confined to the face ; these two cases should, moreover, be joined to the cases of facial hemi-anæsthesia collected above, as we have already compared hemi-anæsthesia and hemi-hyperæsthesia.

Case XXVIII. (Hallopean, *Arch. de Physiol.*, 1876, p. 795.) Serv. Millard, thirty-six years. Diostolic cardiac souffle. June 3rd, violent cephalalgia ; 4th, incomplete left facial paralysis, with paresia of the adductor of right eye. The *sensibility seemed exaggerated in the paralyzed half of the face ;* motility is enfeebled in right arm, this paresis being accompanied with a sensation of numbness ; 6th, paralysis of left face and right limbs more complete ; state of sensibility not noted ; 8th, paralysis extends to the left leg, and is even more complete ; 11th and 12th, dyspnœa. Death.

*Autopsy.*—Upon the floor of the fourth ventricle, to the left of the median furrow, on a line with the *eminentia teres*, an ecchymosis two millimeters in diameter; superjacent to a rounded spot of softening. five millimeters in diameter, situated exactly at the place of origin of the facial abductor. The other parts of the encephalon healthy; obliteration of the extremity of the left vertebral by an embolus from the heart; the clot projecting into the basilar, and prolonged into the infero-posterior cerebellar.

In this case the facial hyperæsthesia occupied the side corresponding to the lesion, and there was a feeling of numbness in the arm of the opposite side. This case of hyperæsthesia then is comparable with the cases of alternate hemi-anæsthesia which we have collected above, and the sensitive troubles of the face should be attributed to the irritation, not as in the preceding cases, solely of the sensitive commissural fasciculi, but of the points of origin of the trigeminal or the peripheric fibers which lie in contact. There was also a lesion of the radical fibers of the trigeminal in one of the experiments of M. Vulpian upon the floor, where he proved hyperæsthesia of the ear of the same side.

In another case, reported by M. Lasègue in his interesting analysis, the hyperæsthesia is still more remarkable from its course, seat, etc. Unfortunately no autopsy.

Case XXIX. (Bastian, *Arch. de Méd.*, 1876, p. 335).—Thirty years. Sharp pains in forehead and left limbs; paralysis at first of the left arm, and then general; face drawn to the right. Loss of hearing, of deglutition, of speech (evident signs of a mesocephalic lesion). After three weeks, voice and hearing return; sensibility absent in limbs of left side, as well as smell of same side; on the contrary, the *left face is hyperæsthesic*. The temperature is generally lowered on left side. There was afterward progressive diminution of the paralytic symptoms, sensitive and motor, of the left side.

We have found no other cases of facial hemi-hyperæsthesia from mesocephalic lesion; but it appears evident to us that more complete reseach would furnish several. Nor have we found a case where the hyperæsthesia instead of affecting the cutaneous sensibility, had affected one special sense only. We should remember, however, that in those of our cases where the anæsthesia, what ever its form, is accompanied by deafness, this deafness has been preceded several times by buzzing in the ears, and even real pain ; and symptoms of the same order are noted in several cases of sclerotic lesion, bringing on the deafness mentioned above, for example, in case II of M. Pierret already cited, etc. There is then an auditory hyperæsthesia of mesocephalic origin ; and consequently it is possible that this symptom may be encountered as an isolated, sensitive trouble.

We terminate here this enumeration of sensitive troubles of mesocephalic origin, troubles, moreover, of which the greater part have already been indicated and studied by Brown-Séquard : we do not pretend to have made it complete and certainly other clinical forms may be realized ; but it suffices us to have called attention to some well defined syndromes in the double point of view, anatomical and clinical ; opposite hemi-anæsthesia, alternate hemi-anæsthesia, general anæsthesia, limited anæsthesia, general hyperæsthesia , hemi-hyperæsthesia, etc.

Although having insisted but little upon the anatomical localization of these sensitive troubles, waiting to collect and point out precisely these facts in the following conclusions, we have not feared to draw sometimes from a small number of cases important deductions ; for unless admitting that the same lesion may determine different effects, for proving a constant relation, a law. it needs not numerous observations, but precise ones.

Unfortunately many of the cases that we have collected are very incomplete, and some, even those for example, where a lesion of the pons nearly median and of little extent has determined a slight hemi-anæsthesia, remain contradictory until new observations more precise permit us to either reject or interpret them ; and in the mean time we must admit, that at the beginning of these somewhat extended researches upon mesocephalic sensitive troubles, we did not expect facts so completely comparable by their lesions and their symptoms. We did not expect to be able to arrive at conclusions certainly incomplete, but we believe them, exactly and sufficiently precise ; and yet should we have only indicated the *desiderata*, this study would have seemed to us sufficiently useful.

(*a.*) To recapitulate, from the point of view of *the anatomical nature of the lesion*, and in order of frequency, the hemorrhagic effusions have determined all the kinds of sensitive troubles enumerated above; tumors have produced only general *anæsthesia*, hemiplegic or limited; vascular obstructions have caused once general anæsthesia, tolerably frequent hyperæsthetic derangement. Finally, in rare cases variable sensitive symptoms, oftenest not well limited, have been produced by lesions of neighboring organs, aneurisms, abscesses, alteration of the membranes; and also diverse lesions of the cerebellum; this last organ not appearing to us to act upon the sensibility except in interesting the mesocephalon.

(*b.*) The seat of the lesion is especially *important: it is this which determines the form, the localization of the sensitive trouble.*

In no case has the lesion been limited to the bulb; nearly always *the pons*, the sensory perceptive organ (Vulpian, Longet), has been interested, alone or conjointly with a neighboring organ.

Sensitive troubles are perhaps more frequent in lesions of the posterior layers of the pons; but the situation of the lesion in a point more or less above, or more or less distant from the median line, is especially important.

An internal lesion of the pons, median, unilateral, bilateral, even extensive, whatever be its level, determines usually only motor symptoms; and it is only when this internal or median lesion occupies the posterior parts especially, and is very extensive in a vertical direction, that general anæsthesia is met with: if this median lesion, of slight extent, but of special nature, irritative, and often then consecutive to a cerebellar lesion, reach the floor of the fourth ventricle, there may be general hyperæsthesia.

A lesion of the external fasciculi of the pons, or of their peduncular prolongations, brings on opposite hemi-anæsthesia of face and limbs, the same external lesion seated a little lower, of the bulb and pons, or more diffuse, occupying nearly one side of the pons, determines alternate hemi-anæsthesia, face of same side, limbs of the opposite side; finally, there are some cases of unilateral, facial or auditory anæsthesia, from limited lesion of bulb and pons of the same side.

An intermediary lesion, often antero-lateral, located towards the middle cerebellar peduncle, extending towards the external fasciculi, without destroying them, produces opposite hemi-hyperæsthesia.

(*c.*) Troubles of the senses, inconstant and variable in our cases, which are very incomplete in this respect, affect especially the hearing, sometimes the taste, whithout it being possible to indicate their relations with such and such anatomico-symptomatological forms. They are often unilateral, and then occupy, according to the height of the lesion, the same side if the lesion is inferior, or the opposite side if it is situated higher up.

Alterations of the trigeminal, or paralysis of the muscles of accommodation, etc., explain certain exceptional cases of derangement of sight or smell.

(*d.*) Paralytic affections of the voluntary muscles have affected the most variable forms, general or bilateral paralysis, simple hemiplegia, or alternate, or ocular, etc., without it being possible to show a constant relation between the form of the motor paralysis or that of the sensory troubles; and thus alternate hemiplegia very frequently has coincided with all the modes of sensory troubles studied above.

(*e.*) The temperature has often been diminished in the anæsthetic parts, whether the anæsthesia be general, hemiplegic or alternate; the state of the temperature has been too rarely, too incompletely noted in hyperæsthesia to make any deductions therefrom.

These conclusions confirm, in many points, results already indicated by the experimentors or anatomists, Foville, Longet, Vulpian, Cl. Bernard, Brown-Séquard, Meynert, Charcot, etc.; and to sum up, we believe we can admit physiologically that the fibers lying in contact with the bulbar nuclei are direct; that other fibers furnished by all the bulbo-medullary nuclei of one side decussate by means of the median gray matter of the pons, with those of the opposite side, going to constitute an external commissural fasciculus of the peduncle and pons.

